# Supplementary material for: Adolescent individual, school, and neighborhood influences on young adult hypertension risk
Source: PLoS One. 2022 Apr 28;17(4):e0266729. doi: 10.1371/journal.pone.0266729 (PMC9049504; doi:10.1371/journal.pone.0266729)
Supplement: S1 Table — (DOCX) [file pone.0266729.s001.docx]

**S1 Table.** Logistic cross-classified multilevel models (CCMM) predicting hypertension based on the 2017 ACC/AHA guideline from individual-, school- and neighborhood-level factors in the National Longitudinal Study of Adolescent to Adult Health, Wave IV (WIV), 2008-2009 (N = 13,926).

| **Hypertension (130/80)** | **Model 1** | **Model 2** | **Model 3** | **Model 4** | **Model 5** |
| --- | --- | --- | --- | --- | --- |
|  | Individual Cross-  Classified | Individual and School  Cross-  Classified | Individual and Neighborhood  Cross-  Classified | Individual, School, and Neighborhood  Cross-  Classified | Individual, School, and Neighborhood  Cross-  Classified |
| **Fixed effect estimates Odds Ratios (95% CI)** | | | | |  |
| Intercept (SE) | 0.23 (0.12,0.43) | 0.32 (0.13, 0.60) | 0.26 (0.18, 0.35) | 0.36 (0.19, 0.53) | 0.17 (0.11, 0.24) |
| **Individual-level** |  |  |  |  |  |
| Age, years (WIV) | 1.08 (1.05,1.10) | 1.07 (1.04, 1.10) | 1.08 (1.06, 1.09) | 1.07 (1.05,1.08) | 1.06 (1.05, 1.08) |
| Female | 0.33 (0.31,0.36) | 0.33 (0.31, 0.36) | 0.33 (0.31, 0.36) | 0.33 (0.31,0.35) | 0.33 (0.30, 0.36) |
| Race/ethnicity |  |  |  |  |  |
| White | REF | REF | REF | REF | REF |
| Black | 1.18 (1.06, 1.31) | 1.26 (1.12, 1.42) | 1.17 (1.02, 1.33) | 1.19 (1.04,1.35) | 1.14 (0.99, 1.29) |
| Asian | 1.00 (0.84, 1.81) | 1.08 (0.89, 1.29) | 1.01 (0.84, 1.20) | 1.05 (0.88,1.26) | 1.20 (0.99, 1.44) |
| Hispanic | 0.89 (0.78, 1.01) | 0.94 (0.82, 1.06) | 0.88 (0.77, 0.99) | 0.92 (0.81,1.04) | 0.85 (0.74, 0.98) |
| Other | 0.91 (0.65, 1.25) | 0.96 (0.67, 1.32) | 0.91 (0.65, 1.23) | 0.92 (0.65,1.25) | 0.88 (0.61, 1.20) |
| Multiracial | 1.01 (0.84, 1.22) | 1.05 0.87, 1.26) | 1.01 (0.84, 1.20) | 1.04 (0.85,1.22) | 0.98 (0.81, 1.18) |
| Parent receipt of public assistance | 1.08 (0.95, 1.23) | 1.06 (0.87, 1.26) | 1.06 (0.93, 1.21) | 1.05 (0.91,1.20) | 1.04 (0.89, 1.21) |
| Parental Education |  |  |  |  |  |
| Less than high school | REF | REF | REF | REF | REF |
| High school graduate / GED | 1.07 (0.94, 1.22) | 1.05 (0.91, 1.19) | 1.07 (0.94, 1.21) | 1.05 (0.91, 1.20) | 1.05 (0.92, 1.20) |
| Some college | 0.97 (0.86, 1.11) | 0.97 (0.84, 1.10) | 0.99 (0.87, 1.13) | 0.98 (0.85, 1.11) | 0.99 (0.86, 1.13) |
| College graduate or beyond | 0.87 (0.77, 0.99) | 0.89 (0.77, 1.02) | 0.92 (0.80, 1.05) | 0.91 (0.78, 1.04) | 0.98 (0.86, 1.13) |
| BMI, kg/m^2^ (WIV) |  |  |  |  |  |
| Under or Normal Weight |  |  |  |  | REF |
| Overweight |  |  |  |  | 1.87 (1.71, 2.04) |
| Obese |  |  |  |  | 3.65 (3.33, 3.98) |
| Unknown |  |  |  |  | 9.69 (1.81, 35.62) |
| Current smoking (WIV) |  |  |  |  |  |
| No |  |  |  |  | REF |
| Yes |  |  |  |  | 1.11 (1.02, 1.20) |
| Unknown |  |  |  |  | 1.32 (0.86, 1.96) |
| **School-level, per 10%** |  |  |  |  |  |
| Percent of students Non-Hispanic White |  | 1.03 (1.01, 1.06) |  | 1.03 (1.00, 1.07) | 1.03 (1.00, 1.06) |
| Percent of parents receiving public assistance |  | 1.01 (0.93, 1.09) |  | 1.01 (0.93, 1.13) | 0.99 (0.90, 1.09) |
| Percent of parents with college degree |  | 0.94 (0.91, 0.97) |  | 0.97 (0.93, 1.02) | 0.97 (0.94, 1.02) |
| **Neighborhood-level, per 10%** |  |  |  |  |  |
| Percent of residents Non-Hispanic White |  |  | 1.00 (0.98, 1.03) | 0.98 (0.95, 1.01) | 1.00 (0.98, 1.03) |
| Percent of residents receiving public assistance |  |  | 1.01 (0.94, 1.10) | 0.99 (0.91, 1.08) | 1.02 (0.94, 1.11) |
| Percent of residents with college degree |  |  | 0.93 (0.89, 0.97) | 0.95 (0.89, 0.99) | 0.97 (0.93, 1.02) |
| **Random effect and variance estimates (95% Credible Interval) [ICC, %]** | | | | |  |
| School | 0.03 (0.01, 0.06) [0.90] | 0.03 (0.01, 0.05) [0.90] | 0.02 (0.01, 0.05) [0.60] | 0.02 (0.00, 0.04) [0.60] | 0.02 (0.01, 0.04) [0.6] |
| Neighborhood | 0.03 (0.01, 0.05) [0.90] | 0.00 (0.00, 0.01) [0.03] | 0.02 (0.01, 0.03) [0.60] | 0.03 (0.01, 0.07) [0.90] | 0.01 (0.00, 0.03) [0.3] |
| Fit statistics (DIC) | 18168.68 | 18167.22 | 18161.98 | 18165.25 | 17323.29 |
