# Supplementary material for: Adolescent individual, school, and neighborhood influences on young adult hypertension risk
Source: PLoS One. 2022 Apr 28;17(4):e0266729. doi: 10.1371/journal.pone.0266729 (PMC9049504; doi:10.1371/journal.pone.0266729)
Supplement: S2 Table — (DOCX) [file pone.0266729.s002.docx]

**S2 Table.** Linear cross-classified multilevel models (CCMM) predicting mean arterial pressure (MAP) from individual-, school- and neighborhood-level factors in the National Longitudinal Study of Adolescent to Adult Health, Wave IV (WIV), 2008-2009 (N = 13,926).

| **Mean Arterial Pressure (mmHg)** | **Model 1** | **Model 2** | **Model 3** | **Model 4** | **Model 5** |
| --- | --- | --- | --- | --- | --- |
|  | Individual Cross-  Classified | Individual and School  Cross-  Classified | Individual and Neighborhood  Cross-  Classified | Individual, School, and Neighborhood  Cross-  Classified | Individual, School, and Neighborhood  Cross-  Classified |
| **Fixed effect estimates β (95% CI)** | | | | |  |
| Intercept (SE) | 87.55 (84.29, 90.76) | 87.93 (84.42, 91.32) | 88.38 (84.92, 91.80) | 88.93 (85.35 92.47) | 85.31 (81.93, 88.77) |
| **Individual-level** |  |  |  |  |  |
| Age, years (WIV) | 0.34 (0.23, 0.45) | 0.33 (0.22, 0.44) | 0.34 (0.23, 0.45) | 0.32 (0.21, 0.43) | 0.27 (0.16, 0.37) |
| Female | -6.48 (-6.81, -6.13) | -6.48 (-6.81, -6.14) | -6.48 (-6.81, -6.15) | -6.49 (-6.82, -6.16) | -6.16 (-6.47, -5.83) |
| Race/ethnicity |  |  |  |  |  |
| Non-Hispanic White | REF | REF | REF | REF | REF |
| Non-Hispanic Black | 1.32 (-6.82, -6.14) | 1.52 (0.95, 2.20) | 1.11 (0.49, 1.74) | 1.21 (0.68, 1.82) | 0.96 (0.35, 1.56) |
| Asian | 1.32 (0.81, 1.82) | 0.54 (-0.35, 2.20) | 0.27 (-0.58, 1.12) | 0.49 (-0.39, 1.38) | 1.13 (0.26, 1.99) |
| Hispanic | -0.45 (-1.03, 0.16) | -0.28 (-0.91, 0.38) | -0.53 (-1.14, 0.09) | -0.33 (-0.97, 0.31) | -0.65 (1.24, -0.06) |
| Other | 0.62 (-0.88, 2.13) | 0.76 (-0.83, 2.33) | 0.06 (-1.02, 2.14) | 0.72 (-0.87, 2.23) | 0.41 (-1.10, 1.92) |
| Multiracial | 0.06 (-0.80, 0.93) | 0.19 (-0.68, 1.08) | 0.02 (-0.83, 0.90) | 0.13 (-0.76, 1.02) | -0.15 (-0.99, 0.68) |
| Parent receipt of public assistance | 0.33 (-0.30, 0.96) | 0.31 (-0.33, 0.94) | 0.26 (-0.37, 0.89) | 0.25 (-0.38, 0.89) | 0.20 (-0.41, 0.82) |
| Parental Education |  |  |  |  |  |
| Less than high school | REF | REF | REF | REF | REF |
| High school graduate / GED | 0.32 (-0.30, 0.96) | 0.29 (-0.32, 0.92) | 0.36 (-0.25, 0.98) | 0.32 (-0.27, 0.94) | 0.19 (-0.41, 0.82) |
| Some college | -0.05 (-0.66, 0.55) | -0.04 (-0.67, 0.59) | 0.05 (-0.59, 0.67) | 0.06 (-0.61, 0.64) | 0.24 (-0.35, 0.83) |
| College graduate or beyond | -0.79 (-1.43, -0.15) | -0.70 (-1.32, -0.06) | -0.58 (-1.22, 0.06) | -0.60 (-1.25, 0.05) | 0.06 (-0.53, 0.65) |
| Anti-hypertensive medications | 6.06 (5.16, 6.94) | 6.04 (5.15, 6.94) | 6.03 (5.13, 6.93) | 6.01 (5.10, 6.91) | 4.57 (3.71, 5.44) |
| BMI, kg/m^2^ (WIV) |  |  |  |  |  |
| Under or Normal Weight |  |  |  |  | REF |
| Overweight |  |  |  |  | 3.54 (3.13, 3.96) |
| Obese |  |  |  |  | 7.13 (6.74, 7.51) |
| Unknown |  |  |  |  | 11.00 (5.13, 16.76) |
| Current smoking (WIV) |  |  |  |  |  |
| No |  |  |  |  | REF |
| Yes |  |  |  |  | 0.98 (0.64, 1.33) |
| Unknown |  |  |  |  | 0.35 (-1.47, 2.18) |
| **School-level, per 10%** |  |  |  |  |  |
| Percent of students Non-Hispanic White |  | 0.09 (-0.04, 0.22) |  | 0.14 (-0.01, 0.29) | 0.12 (-0.01, 0.26) |
| Percent of parents receiving public assistance |  | -0.09 (-0.52, 0.35) |  | -0.09 (-0.55, 0.36) | -0.16 (0.60, -0.17) |
| Percent of parents with college degree |  | -0.20 (-0.39, 0.01) |  | -0.13 (-0.36, 0.08) | -0.06, (0.26, -0.06) |
| **Neighborhood-level, per 10%** |  |  |  |  |  |
| Percent of residents Non-Hispanic White |  |  | -0.04 (-0.16, 0.07) | -0.09 (-0.22, 0.03) | -0.04 (-0.16, -0.04) |
| Percent of residents receiving public assistance |  |  | 0.09 (-0.27, 0.45) | 0.02 (-0.37, 0.42) | 0.09 (-0.27, 0.09) |
| Percent of residents with college degree |  |  | -0.01 (-0.21, 0.19) | -0.17 (-0.38, 0.05) | -0.01 (-0.21, -0.01) |
| **Random effect and variance estimates (95% Credible Interval) [ICC, %]** | | | | |  |
| Individual | 100.21 (97.90, 102.58) [98.9] | 100.10 (97.72, 102.51) [98.9] | 100.11 (97.72, 102.51) [98.2] | 100.19 (97.88, 102.61) [99.1] | 91.72 (89.61, 93.93) [99.2] |
| School | 1.06 (0.57, 1.69) [1.05] | 0.96 (0.50, 1.54) [0.95] | 0.89 (0.45, 1.47) [0.87] | 0.90 (0.45, 1.49) [0.89] | 0.71 (0.34, 1.20) [0.6] |
| Neighborhood | 0.04 (0.00, 0.29) [0.04] | 0.17 (0.01, 0.66) [0.17] | 0.18 (0.01, 0.66) [0.88] | 0.05 (0.01, 0.20) [0.05] | 0.06 (0.00, 0.27) [0.1] |
| Fit statistics (DIC) | 103647.14 | 103646.40 | 103646.32 | 103646.39 | 102421.50 |
