# Supplementary material for: Adolescent individual, school, and neighborhood influences on young adult hypertension risk
Source: PLoS One. 2022 Apr 28;17(4):e0266729. doi: 10.1371/journal.pone.0266729 (PMC9049504; doi:10.1371/journal.pone.0266729)
Supplement: S3 Table — (DOCX) [file pone.0266729.s003.docx]

**S3 Table.** Crude cross-classified multilevel models (CCMM) predicting hypertension, systolic blood pressure, diastolic blood pressure, mean arterial pressure (MAP) from individual-, school- and neighborhood-level factors in the National Longitudinal Study of Adolescent to Adult Health, Wave IV, 2008-2009 (N = 13,926).

|  |  | **Model 1** | **Model 2** | **Model 3** | **Model 4** |
| --- | --- | --- | --- | --- | --- |
|  |  | Individual-only | Individual  and  School | Individual  and  Neighborhood | Individual, School,  and Neighborhood |
| Hypertension  (140/90) | **Fixed effect intercept** **estimate (95% CI)** | -1.34 (-1.38, -1.31) | -1.36 (-1.42, -1.31) | -1.34 (-1.38, -1.30) | -1.37 (-1.43, -1.31) |
|  | **Random effect variance parameter estimates (95% CI) [ICC]** | | | | |
|  | Individual | - | - | - | - |
|  | School | - | 0.05 (0.03, 0.08) | - | 0.05 (0.02, 0.08) [1.5] |
|  | Neighborhood | - | - | 0.01 (0.00, 0.01) | 0.01 (0.00, 0.02) [0.3] |
|  | **DIC** | 14200.78 | 14145.14 | 14200.03 | 14148.88 |
| Hypertension  (130/80) | **Fixed effect intercept** **estimate (95% CI)** | 0.05 (0.01, 0.08) | 0.03 (-0.03, 0.08) | 0.02 (-0.02, 0.06) | 0.03 (-0.03, 0.08) |
|  | **Random effect variance parameter estimates (95% CI) [ICC]** | | | | |
|  | Individual | - | - | - | - |
|  | School | - | 0.05 (0.03, 0.08) | - | 0.04 (0.02, 0.07) [1.2] |
|  | Neighborhood | - | - | 0.06 (0.02, 0.09) | 0.02 (0.01, 0.05) [0.6] |
|  | **DIC** | 19279.73 | 19196.78 | 19231.24 | 19188.16 |
| Systolic  Blood  Pressure | **Fixed effect intercept estimate** **(95% CI)** | 124.5 (124.3, 124.7) | 124.4 (124.0, 124.8) | 124.35 (124.1, 124.6) | 124.4 (124.1, 124.8) |
|  | **Random effect variance parameter estimates (95% CI) [ICC]** | | | | |
|  | Individual | 185.5 (181.2, 189.9) | 183.3 (179.1, 187.6) | 183.1 (178.7, 187.5) | 182.9 (178.6, 187.2) [98.5] |
|  | School | - | 2.19 (1.27, 3.38) | - | 1.98 (1.08, 3.15) [1.1] |
|  | Neighborhood | - | - | 3.52 (1.36, 3.92) | 0.67 (0.16, 1.72) [0.4] |
|  | **DIC** | 112128.88 | 112042.24 | 112079.44 | 112039.90 |
| Diastolic  Blood  Pressure | **Fixed effect intercept estimate** **(95% CI)** | 79.04 (78.9. 79.2) | 78.9 (78.6, 79.2) | 78.9 (78.7, 79.1) | 78.9 (78.6, 79.2) |
|  | **Random effect variance parameter estimates (95% CI) [ICC]** | | | | |
|  | Individual | 103.2 (100.8, 105.6) | 101.6 (99.2, 104.0) | 101.7 (99.2, 104.1) | 101.3 (98.9, 103.7) [98.2] |
|  | School | - | 1.54 (0.96, 2.28) | - | 1.47 (0.91, 2.19) [1.4] |
|  | Neighborhood | - | - | 1.57 (0.88, 2.38) | 0.39 (0.08, 0.89) [0.4] |
|  | **DIC** | 103968.85 | 103836.43 | 103909.22 | 103831.16 |
| Mean  Arterial  Pressure  (MAP) | **Fixed effect estimate intercept** | 94.18 (94.0, 94.4) | 94.1 (93.8, 94.4) | 94.07 (93.85, 94.28) | 94.06 (93.77, 94.36) |
|  | **Random effect variance parameter estimates (95% CI) [ICC]** | | | | |
|  | Individual | 114.0 (111.4, 116.8) | 112.4 (109.7, 115.0) | 112.33 (109.65, 115.03) | 112.07 (109.42, 114.75) [98.2] |
|  | School | - | 1.67 (1.03, 2.48) | - | 1.56 (0.95, 2.35) [1.4] |
|  | Neighborhood | - | - | 1.80 (1.02, 2.71) | 0.44 (0.09, 1.00) [0.4] |
|  | **DIC** | 105363.60 | 105239.18 | 105301.18 | 105234.98 |

Abbreviations: CI – credible interval; DIC – deviance information criteria.
